# The relationship between proactive personality and migrant workers’ perception of technical unemployment risk under the impact of artificial intelligence in China

**DOI:** 10.3389/fpsyg.2025.1474639

**Published:** 2025-03-19

**Authors:** Guanglu Xu, Ming Xue

**Affiliations:** ^1^School of Business, Nanjing University of Information Science & Technology, Nanjing, China; ^2^School of Business Administration, Shanghai Lixin University of Accounting and Finance, Shanghai, China

**Keywords:** proactive personality, AI learning, AI self-efficacy, perception of technical unemployment risk, migrant workers

## Abstract

**Introduction:**

Under the background of rapid progress of artificial intelligence (AI) technology, migrant workers are at a high risk of becoming unemployed due to their low levels of human capital. As for the influencing factors of unemployment risk perception, individual characteristics are an important one. However, there remain few studies exploring the impact of personality on the perception of technical unemployment risk among migrant workers under the impact of AI. Therefore, this study examines the relationship between proactive personality and migrant workers’ perception of technical unemployment risk, focusing on the mediating roles of AI learning and AI self-efficacy.

**Methods:**

This study collected data through the research platform “Credamo”. A total of 551 questionnaires were recovered in this survey, of which 446 valid questionnaires were reserved for research.

**Results:**

The research results show that: proactive personality was negatively correlated with migrant workers’ perception of technical unemployment risk; AI learning and AI self-efficacy mediated this relationship independently, and played a chain-mediating role in this relationship.

**Discussion:**

The present study contributes the perception of technical unemployment risk literature by highlighting the impact of proactive personality and the chain mediating role of AI learning and AI self-efficacy in a sample of Chinese migrant workers. The empirical results of this paper are considered as helpful for organizations to formulate the policies aimed at alleviating migrant workers’ perception of technical unemployment risk under the impact of AI technology.

## 1 Introduction

The concept of artificial intelligence (AI), which was first proposed at the Dartmouth Conference in 1956, refers to “the capability of a machine to imitate intelligent human behavior” or “an agent’s ability to achieve goals in a wide range of environments” (Aghion et al., 2018; Chen and Xu, 2018). With the development of computer technology, AI technology entered a stage of rapid development in the 1990s (Hui and Jiang, 2020). At the same time, it also had an impact on the society in all aspects. The academic community has begun to widely discuss the impact of AI technology on labor employment. It is argued in many studies that the development of AI technology will lead to the replacement of existing occupations to varying degrees. In Japan, 55% of jobs will be replaced by AI (David, 2017). In the United States, the figure is 47% (Frey and Osborne, 2017), and in China, the figure is 65.58% (Chen and Xu, 2018). Migrant workers are those who have a rural household registration, who are mainly engaged in non-agricultural work (He, 2024). According to China’s National Bureau of Statistics, there will be 299 million migrant workers across the country in 2024. The main motivation for migrant workers to move to cities is personal development. Without urban household registration, employment has become an important way for workers migrating from rural areas to integrate into the city (Liang and Zhang, 2018), and stable employment is crucial for these migrant workers, which is an important factor affecting their mental health (Nie and Feng, 2013). Meanwhile, migrant workers are a vulnerable group in the society due to their low level of human capital, and their ability to resist unemployment risks is weak (Yang and Shao, 2021). Therefore, it is necessary to discuss the unemployment risk of migrant workers under the impact of AI.

Unemployment risk refers to the possibility of unemployment of people who are able to work (Wang, 2001). However, risk exists objectively, but everyone’s cognition is different, for which different people take different attitudes towards risk (Zheng and Zhang, 2021). Therefore, to investigate the impact of unemployment risk on individuals, it is necessary to examine the individual perception of unemployment risk. Unemployment risk perception refers to workers’ perception and understanding of various external objective risks that may lead to unemployment, mainly including policy unemployment risk perception, individual unemployment risk perception and technical unemployment risk perception (Li et al., 2021). This paper focuses mainly on investigating the perception of unemployment risk presented by AI technology. Therefore, it belongs to the perception of technical unemployment risk, that is, the perception of unemployment risk posed by technological factors. The perception of unemployment risk tends to induce a series of negative behavioral reactions and attitudes of individuals, such as reduced job satisfaction and job identity, etc. These consequences undermine the sustainable development of migrant workers’ career. In serious cases, it even affects mental health and leads to various psychological problems such as depression (Laine et al., 2009; Lange, 2013). Therefore, when the change of AI technology is implemented, it is an extremely urgent research topic to investigate the factors relating to the perception of technical unemployment risk among migrant workers, a vulnerable group, and to formulate the policies to intervene.

As for the influencing factors of unemployment risk perception, individual characteristics are an important one (Li et al., 2021). For example, an individual’s education level, occupational license obtained, industry and employment type are all the key factors (Wang et al., 2021). The knowledge and skills acquired from past work experience are also the significant factors influencing the perception of unemployment risk (Zheng and Zhang, 2021). However, there remain few studies exploring the impact of personality on the perception of technical unemployment risk among migrant workers under the impact of AI. The Social Cognition Theory holds that human activity is determined by behavior, cognitive and other personal factors, and environmental events; people are both products and producers of their environment (Wood and Bandura, 1989). In other words, although the external environment exerts influence on individual psychology and behavior, individuals can also take measures to change their external environment. Proactive personality is an individual’s tendency to take initiative and influence the environment (Bateman and Crant, 1993). Studies have also shown that proactive personality is related to a variety of desirable individual outcomes, including objective career success (e.g., salary and promotions) and job performance (Yang and Chau, 2016), conducting career planning, pursuing opportunities for self-improvement and persisting in face of career obstacles (Seibert et al., 1999). Furthermore, the perceived employability of individuals can be improved (Guilbert et al., 2018), thereby reducing the perceived risk of technological unemployment. Therefore, proactive personality may be an important factor influencing migrant workers’ perceptions of technical unemployment risk under the influence of AI.

The development of AI poses a major threat to the career development of workers. To mitigate this threat, individuals need to change their knowledge and skill structures in time (Wang et al., 2024). In other words, they need to engage in career-related continuous learning, which refers to the self-initiated, continuous, planned and active, formal or informal accumulation of knowledge and skills for the current or future career development (Kuznia et al., 2010). AI learning refers to the continuous learning of individuals to accumulate AI-related knowledge and skills, which is an effective way to successfully cope with threats to career development. According to the Social Cognition Theory (Wood and Bandura, 1989), employees with strong proactive personality traits tend to devote themselves to and participate in some self-learning activities to increase their knowledge and improve their skills (Zhan et al., 2018). As they acquire more knowledge related to AI, their professional skills will be enhanced, improving their long-term employability (Hall, 1996; Bednall and Sanders, 2017). Moreover, they can objectively view the impact of external threats on themselves, thus reducing their risk perception (Zhou and Tang, 2017). Therefore, AI learning may be a mediator between proactive personality and the risk perception of technical unemployment.

Self-efficacy refers to people’s belief in their abilities to achieve behavioral goals in a particular area (Brown et al., 2006). AI self-efficacy reflects an individual’s confidence in taking action to deal with the threats that the AI technology poses to their career development. Individuals with stronger proactive personalities are more likely to actively improve or change their environment (Crant and Bateman, 2000). Under the situation of AI threats, individuals can actively take measures to weaken the threats of AI to their career development. The experience of successfully dealing with threats is an important source of self-efficacy, which will increase their self-efficacy (Wood and Bandura, 1989). According to the Social Cognitive Theory, human beings are active behavior managers, and human agency depends on their self-efficacy (Bandura and Locke, 2003). We can therefore speculate that individuals with higher levels of AI self-efficacy are more confident in their abilities to manage the impact of AI on their careers. Even if they encounter short-term impact, it is more likely that they can achieve career success in the long run (Seibert et al., 1999). Thus, the risk perception of technical unemployment is lower. Therefore, AI self-efficacy may be a mediator between proactive personality and the risk perception of technical unemployment.

According to the Social Cognitive Theory, important ways of learning include the accumulation of experience acquired through personal practice and imitating others, both of which are important sources for the improvement of self-efficacy (Wood and Bandura, 1989). On the one hand, learning can help individuals master the knowledge and skills related to AI, through which individuals can improve their competence (Gomez-Baya and Lucia-Casademunt, 2018), thus enhancing their AI self-efficacy. On the other hand, when employees learn AI knowledge and skills in an organization, the interaction among various subjects in the learning process sets a model for individuals, which can then help them improve AI self-efficacy (Wood and Bandura, 1989). Combined with the above analysis, proactive personality promotes the process of AI learning for migrant workers. AI learning improves the AI self-efficacy of migrant workers, while AI self-efficacy is negatively correlated to their perception of technical unemployment risk. Therefore, AI learning and AI self-efficacy may play a chain-mediating role in the relationship between proactive personality and the risk perception of technical unemployment.

Following the above theoretical analysis, the mediating mechanism between proactive personality and migrant workers’ perception of technical unemployment risk is established based on social cognitive theory. In summary, this study poses the following questions: (i) Does proactive personality have relationship with the risk perception of technical unemployment? (ii) Do AI learning and AI self-efficacy mediate the relationship between proactive personality and the risk perception of technical unemployment? (iii) Do AI learning and AI self-efficacy play a chain-mediating role in the relationship between proactive personality and the risk perception of technical unemployment? The research results of this paper are beneficial for organizations to formulate the policies purposed to reduce the perception of technical unemployment risk among migrant workers, help migrant workers adapt to the organizational AI technological change, and then promote the smooth implementation of AI technological change in organizations.

## 2 Research hypotheses

### 2.1 Proactive personality and the risk perception of technical unemployment

After the application of the AI technology, the internal position setting will inevitably change, traditional repetitive work will be replaced, and more jobs requiring new skills related to AI will appear (Xie et al., 2019). This phenomenon is known as technological unemployment, that is, the unemployment caused by technological development (Chen and Xu, 2018). However, external risks are often objective, while risk perception means subjective judgments made by individuals (Zheng and Zhang, 2021). Migrant workers are mostly engaged in routine work, and they are more pessimistic about their job prospects and have a greater perception of unemployment risk (Wang et al., 2021). According to the Social Cognition Theory (Wood and Bandura, 1989), human activities are determined by behavior, cognitive and other personal factors, and environmental events; people are both products and producers of their environment. In other words, although the external environment exerts influence on individual psychology and behavior, individuals can also take measures to change their external environment and reduce the threats posed by the external environment to themselves. The application of AI will dramatically change migrant workers’ working environment, which will enhance their perception of technical unemployment risk. In this process, personality will influence the environment, mitigate the threats of AI reforms for migrant workers, and subsequently alleviate their perception of technical unemployment risk. Specifically, the different personalities of migrant workers will lead to the variations in their attitudes taken towards risks, thus leading to their difference in their risk perception.

A proactive personality is an individual’s tendency to take initiative and influence the environment (Bateman and Crant, 1993). When an AI reform is implemented, many work contents have changed, and employees face the restructuring of their career development. Mastering AI-related skills can expand the range of job mobility, which in turn reduces the risk of unemployment (Duan and Guo, 2018). What is particularly important in this process is whether individuals can recognize this opportunity and take actions to carry out a vocational skill transfer. Studies have shown that employees with strong proactive personality are more likely to identify and pursue career opportunities, engage in career management activities, and achieve career success (Yang and Chau, 2016). Therefore, after the application of AI by enterprises, migrant workers with strong proactive personality are more able to adapt to this change proactively. Mao and Hu found out that with the change of AI, employees needed to seek new job opportunities by actively improving their skills and completing a functional conversion (Mao and Hu, 2020). Therefore, when enterprises apply AI technology, migrant workers with strong proactive personality can improve their skills and complete their skill transformation promptly, which makes it easier to seek new job opportunities and lower the perception of technical unemployment risk.

Therefore, this paper proposes the following hypothesis:

*Hypothesis H1*: Proactive personality is negatively associated with the perception of technical unemployment risk.

### 2.2 The mediating role of AI learning

With the popularization of the AI technology in enterprises, some positions will be gradually replaced by intelligent machines, and migrant workers will face huge uncertainties in their career development. According to Social Cognitive Theory, the environment will affect the individuals, and at the same time, the individuals take actions to change the environment (Wood and Bandura, 1989). In the context of AI revolution, in order to reduce the impact of external environment on themselves, individuals need to change their knowledge and skills structure in time (Wang et al., 2024). AI Learning is an effective way to achieve this goal. In this process, the individuals’ self-control ability plays a great role (Qi et al., 2023).

Proactive personality refers to the stable tendency of an individual to take actions to influence the environment in which they live (Bateman and Crant, 1993). Employees with strong proactive personality traits tend to devote themselves to and participate in some self-learning activities for increasing their knowledge and improving skills, which is a preparation made for influencing and changing the environment (Zhan et al., 2018). Therefore, it can be speculated that under the impact of AI, migrant workers with strong proactive personality traits are more able to learn AI-related knowledge and skills. Under the impact of the AI technology, learning is effective for reducing the perception of technical unemployment risk. The mechanism behind it mainly includes two aspects. Firstly, when accumulating AI-related knowledge and skills through learning, migrant workers will have more promotion opportunities, which could lower their perception of technical unemployment risk (Chen et al., 2022). Studies have also shown that learning can help employees expand their knowledge and skills, enhance their adaptability to a rapidly-changing environment, and thus improve their long-term employability (Hall, 1996; Bednall and Sanders, 2017), which leads to a lower perception of technical unemployment risk. Secondly, learning can help migrant workers master the knowledge of risks related to occupational threats posed by the development of AI. According to Risk Perception Theory, the understanding of risk-related knowledge can help individuals objectively view the impact of external threats on themselves, thus reducing their risk perception (Zhou and Tang, 2017).

From the above discussion, it can be found out that under the impact of AI, proactive personality will prompt migrant workers to proactively learn AI-related knowledge and skills. In turn, learning AI-related knowledge and skills will reduce their perception of technical unemployment risk. Therefore, this paper proposes the following hypothesis.

*Hypothesis H2*: AI learning plays a mediating role between proactive personality and migrant workers’ perception of technical unemployment risk.

### 2.3 The mediating role of AI self-efficacy

Self-efficacy refers to people’s belief in their abilities to achieve behavioral goals in a particular area (Brown et al., 2006). Generally speaking, self-efficacy tends to be directed towards specific task situations. Compared with the general self-efficacy, the self-efficacy directed towards specific task situations has a higher predictive power for individual behavior (Bandura, 1999). AI self-efficacy reflects the confidence of individuals in taking measures to deal with the threats posed by the AI technology to their career development. Individuals with a stronger proactive personality are more inclined to actively improve or change their environment, and are more willing to actively cope with and change the status quo, but are less willing to passively adapt to it (Crant and Bateman, 2000). This personality trait determines that in the face of AI technological changes, they have stronger confidence to adapt to the impact of technological changes on them, and AI self-efficacy is stronger. Meanwhile, the experience of successfully dealing with AI threats is an important source of self-efficacy, which will increase their self-efficacy (Wood and Bandura, 1989). When problems encountered by individuals are dealt with, positive coping strategies can help them improve their self-efficacy in problem-solving (Benatov, 2019). Existing studies have also confirmed the positive relationship between proactive personality and self-efficacy (Seibert et al., 1999; Shang and Gan, 2009; Li et al., 2017).

According to the Social Cognitive Theory, human beings are active behavior managers, and human agency depends on self-efficacy (Bandura and Locke, 2003). Research has consistently revealed that an individual’s self-efficacy expectations are associated with increased efforts, persistence, and goal-directed activities (Bandura, 1986). Therefore, it can be inferred that, when individual have a higher level of AI self-efficacy, they are more confident to cope with the impact of AI on their career. Even if they encounter short-term impact, it is more likely that they can achieve career success in the long run (Seibert et al., 1999). In other words, in threat situations caused by the AI technology, individuals with a high AI self-efficacy can actively respond to threats, who will not possibly be replaced by AI technology, but may also seize the opportunities brought by it, achieve greater career development, and even reduce their risk perception of technical unemployment (Nursey-Bray et al., 2012; Maltby et al., 2021). According to the above analysis, proactive personality is significantly correlated with the AI self-efficacy of migrant workers in the process of coping with AI. In other words, AI self-efficacy can reduce migrant workers’ perception of technical unemployment risk. Therefore, this paper proposes the following hypothesis.

*Hypothesis H3*: AI self-efficacy plays a mediating role between proactive personality and migrant workers’ perception of technical unemployment risk.

### 2.4 The chain mediating role of AI learning and AI self-efficacy

According to the Social Cognitive Theory, important ways of learning include the accumulation of experience acquired through personal practice and imitating others, both of which are important sources for the improvement of self-efficacy (Wood and Bandura, 1989). The mechanism that lies behind this is reflected in the following aspects in particular. Firstly, learning can help individuals master the knowledge and skills related to AI, through which individuals can improve their competency (Gomez-Baya and Lucia-Casademunt, 2018), thus enhancing their confidence in dealing with the AI technology. Secondly, from the perspective of Social Cognitive Theory, the cognition and behavior of employees are influenced by witnessing various interpersonal activities within an organization (Zhan et al., 2019). When employees learn AI knowledge and skills in the organization, the interaction among various subjects in the learning process sets a model for individuals. According to Social Cognitive Theory, role models can help individuals improve their sense of AI self-efficacy (Wood and Bandura, 1989). So, in this process, individuals can establish confidence in mastering the AI technology. According to the above analysis, proactive personality promotes the AI learning process of migrant workers. AI learning improves their AI self-efficacy, while AI self-efficacy is negatively correlated to their perception of technical unemployment risk. Therefore, this paper proposes the following hypothesis.

*Hypothesis H4*: AI learning and AI self-efficacy play a chain-mediating role in the relationship between proactive personality and migrant workers’ perception of technical unemployment risk.

**Figure 1:**
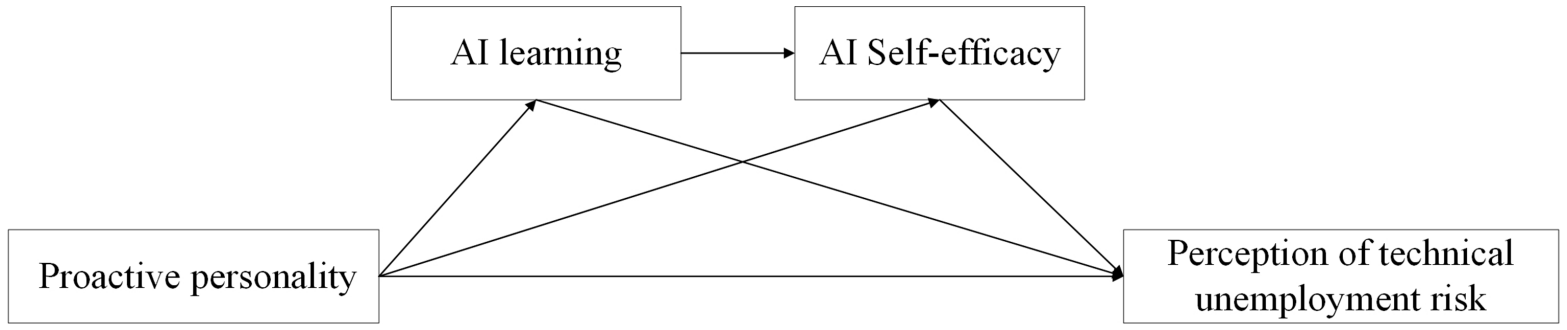
Conceptual model.

The conceptual model constructed in this study is shown in Figure 1.

## 3 Method

### 3.1 Participant and recruitment

This study collected data through the research platform Credamo. Credamo’s own sample bank consists of more than 2.8 million samples from 3,000 companies across the country, which are paid to conduct research. When sample survey is conducted, the platform can set sample characteristics to focus on specific groups for survey. In this paper, the sample was set as the rural household registration with engagement in non-agricultural work. A total of 600 questionnaires were released in this survey, of which 551 questionnaires were recovered. Since our research is about the perception of migrant workers’ unemployment risk in the context of AI, migrant workers should have some understanding of it. So, in the course of this survey, we asked them if their companies were using AI devices in their production and operations, and we did not use questionnaires in which the respondents’ companies were not using AI devices. Also, various invalid samples such as missing and contradictory options were removed. In the end, 446 samples remained. Of the respondents, 58.1% were male. These respondents were mainly aged between 19 and 67, with those aged between 19 and 45 accounting for 89%. Married people account for 71.1%. University graduates accounted for 37.2%, Master and Ph.D. accounted for 4.5% and others for 58.3%. These respondents came from a variety of occupations, including production workers, clerical/office staff, salespersons, etc.

### 3.2 Measures

*Proactive personality*: The scale developed by Wu et al. (2014) was adopted. The scale has three forward items (e.g., “In the face of sharp criticism, I was able not to lose heart”), and one reverse item (e.g., “In the face of sharp criticism, I was able not to lose heart”). In this study, Cronbach’s *α* = 0.706.

*AI self-efficacy*: The scale used by Arogundade and Arogundade (2015) was adopted and revised in the context of AI development. The scale contains three items, one of which is “I am confident in my ability to cope with the change of AI.” In this study, Cronbach’s α = 0.722.

*AI learning*: AI learning was measured by a question raised to the respondents, “Have you acquired knowledge and skills related to AI technologies in any way during the past 3 months?” From never to always, the value ranging from 1 to 5 was assigned.

*Perception of technical unemployment risk*: The scale developed by Hovick et al. (2011). was used for reference, and appropriate revisions were made under the research background of this paper. The scale has a total of three items, one of which is: “I am very likely to lose my job because of the development of AI.” Cronbach’s *α* = 0.865.

*Control variables*: To avoid other variables interfering with the relationship among the core variables in this article, individual characteristic variables were taken as control variables, such as age, gender, and marriage. According to a study of Zheng and Zhang (2021), human capital is an important variable affecting the perception of unemployment risk. Referring to Wen and Xiang (2024), we measure human capital using years of education and participation in skill training. In this paper, education level is converted into years of education, primary school and below = 6; junior high school = 9; general high school/secondary school/technical school/vocational high school =12; college graduate =15; university graduate = 16; master =19; Ph.D. =23. Participation in skills training is measured by the question “Have you participated in free vocational training provided by the Government,” Yes =1, No =0. Liu and Ding (2023) believed that income and whether a labor contract is signed will affect the perception of unemployment risk. Therefore, this paper takes labor contract signing and income as control variables; labor contract signing is measured by the question “The type of labor contract you have signed with your employer is?” A labor contract with and without a fixed term =1, no =0; income is measured by the question “How much did you earn last month?” According to Xu (2022), different occupations are threatened differently by the AI technology, so occupations are also set as control variable, which are divided into seven categories, namely physical occupations (production workers, flexible employment), transactional occupations (administrative/logistics staff, clerical/office staff), marketing occupations (salespersons, marketing/ PR, customer service), professional occupations (human resources management, finance/auditing, educators, consultants/consultants, professionals (such as accountants, lawyers, architects, health care workers, journalists)), technical research and development occupations (technology/R&D), management occupations (management), and others. Since occupation is a categorical variable, so it is necessary to transform the categorical variable into a set of corresponding dummy variables. This paper takes managerial occupation as the reference and includes six other types of dummy variables into the model.

### 3.3 Data processing

The SPSS 24.0 and MPLUS8.3 software were used in the analysis. Specifically, MPLUS8.3 is used to carry out confirmatory factor analysis; SPSS PROCESS macro 3.4 is used to examine the mediating role of AI learning and AI self-efficacy, and chain-mediating role of AI learning and AI self-efficacy.

## 4 Results

### 4.1 Confirmatory factor analysis

In order to further verify the discriminant validity of each variable, confirmatory factor analysis was conducted to perform test. There are three core latent variables concerned in this paper: proactive personality, AI self-efficacy and perception of technical unemployment risk. Confirmatory factor analysis was performed using MPLUS software. The analytical results are shown in Table 1. According to the results of data analysis, the three-factor model shows the best fit with the samples (CFI = 0.961, TLI = 0.945, RMSEA =0.063, SRMR = 0.048). This result confirms the discriminative validity between these variables, which can be further studied.

**Table 1:** Confirmatory factor analysis

Model	Model Description	χ2	df	△χ2(△df)	CFI	TLI	RMSEA	SRMR
1	Three-factor model	89.088	32		0.961	0.945	0.063	0.048
2	Two-factor model	186.832	34	97.744(2)***	0.896	0.862	0.100	0.063
3	Two-factor model	359.815	34	270.727(2)***	0.777	0.705	0.147	0.113
4	Two-factor model	377.988	34	288.900(2)***	0.765	0.689	0.151	0.121
5	Single-factor model	561.715	35	472.627(3)***	0.640	0.537	0.184	0.128

1. It is a hypothetical model. 2. AI self-efficacy and proactive personality are combined as one factor. 3. AI self-efficacy and perception of technical unemployment risk are combined as one factor. 4. proactive personality and perception of technical unemployment risk are combined as one factor; 5. All variables are combined as one factor. ∆χ^2^ test is relative to Model 1. *** represents *p* < 0.001.

### 4.2 Common method variance

The three key core latent variables used in this paper, which are proactive personality, AI efficacy and perception of technical unemployment risk, come from the same source. In order to prevent the common method variance from interference with the reliability of the research results, Harman’s single factor test was used this study to test the common method variance. The test results show that the proportion of the total variance in the first factor is 37.632%, which is lower than 40%, indicating that the common method variance of data is insignificant.

### 4.3 Descriptive statistics and correlation analysis

Means, standard deviations and correlations between the research variables are presented in Table 2. It can be seen from Table 2 that proactive personality is positively correlated with AI learning (r = 0.384, *p* < 0.01), positively correlated with AI self-efficacy (*r* = 0.459, *p* < 0.01), and negatively correlated with perception of technical unemployment risk (*r* = −0.316, *p* < 0.01). AI learning is positively correlated with AI self-efficacy (*r* = 0.385, *p* < 0.01), and negatively correlated with perception of technical unemployment risk (*r* = −0.199, *p* < 0.01). AI self-efficacy is negatively correlated with the perception of technical unemployment risk (*r* = −0.363, *p* < 0.01).

**Table 2:** Descriptive statistics of variables and correlation matrix.

	M	SD	1	2	3	4	5	6	7	8	9	10
1. Age	32.72	9.183										
2. Marriage	0.710	0.454	0.488^**^									
3. Gender	0.580	0.494	0.070	−0.021								
4. Years of education	14.121	2.540	−0.252^**^	−0.116^*^	0.041							
5. Participation in skills training	0.530	0.499	0.002	0.088	0.098^*^	0.016						
6. Labor contract signing	0.930	0.255	−0.081	−0.058	0.089	0.204^**^	0.098^*^					
7. Wage	0.780	0.980	0.150^**^	0.103^*^	0.138^**^	0.207^**^	0.082	0.080				
8. Proactive personality	3.837	0.550	0.007	0.194^**^	0.057	0.112^*^	0.235^**^	0.080	0.102^*^			
9. AI learning	3.190	0.854	0.029	0.195^**^	0.137^**^	0.177^**^	0.277^**^	0.133^**^	0.144^**^	0.384^**^		
10. AI self−efficacy	3.937	0.650	−0.047	0.202^**^	0.093	0.177^**^	0.194^**^	0.082	0.070	0.459^**^	0.385^**^	
11. Perception of technical unemployment risk	2.591	1.001	−0.078	−0.121^*^	−0.023	−0.187^**^	−0.145^**^	−0.088	−0.127^**^	−0.316^**^	−0.199^**^	−0.363^**^

* *p* < 0.05; ** *p* < 0.01; two-tailed tests. SD = standard deviation. Marriage is coded 1= married and 0=unmarried; Gender is coded 1 = male and 0 = female.

**Table 3:** Multiple linear regression analysis results.

	Model 1:PTUR	Model 2:AL	Model 3:PTUR	Model 4:AS	Model 5:PTUR
Age	−0.064	−0.020	−0.065	−0.118*	−0.094
Gender	0.009	0.075	0.012	0.074	0.028
Marriage	−0.048	0.131*	−0.044	0.195***	0.001
Years of education	−0.083	0.121*	−0.079	0.107*	−0.056
Participation in skills training	−0.074	0.181***	−0.067	0.071	−0.056
Labor contract signing	−0.042	0.066	−0.040	0.016	−0.038
Wage	−0.016	0.041	−0.015	−0.018	−0.021
Management occupations					
Physical occupations	0.262***	−0.042	0.261***	−0.027	0.255***
Transactional occupations	−0.042	−0.050	−0.044	−0.035	−0.051
Marketing occupations	0.066	0.040	0.068	0.061	0.082
Professional occupations	−0.012	−0.064	−0.014	0.032	−0.004
Technical research and development occupations	−0.063	0.030	−0.062	0.034	−0.055
Others	0.019	−0.012	0.018	−0.016	0.015
Proactive personality	−0.241***	0.275***	−0.232***	0.379***	−0.145**
AI learning			−0.034		
AI self-efficacy					−0.253***
R^2^	0.226	0.247	0.227	0.275	0.272
F	8.978***	10.072***	8.402***	11.672***	10.716***

* *p* < 0.05; ** *p* < 0.01; *** *p* < 0.001; two-tailed tests. PTUR=perception of technical unemployment risk; AL=AI learning; AS=AI self-efficacy.

### 4.4 Hypotheses testing

In this paper, multiple linear regression was first performed to test Hypothesis H1, Hypothesis H2 and Hypothesis H3, the results of which are shown in Table 3. After controlling for the relevant variables, Proactive personality is negatively associated with migrant workers’ perception of technical unemployment risk (Model M1, *β* = −0.241, *p* < 0.001). Thus, Hypothesis H1 is supported. This result shows that although a large proportion of jobs will be replaced under the threat of AI, as long as migrant workers can actively respond and take measures to change the environment they live in, the impact of AI on their career development can be reduced. According to the procedure proposed by Baron and Kenny to test mediating effect (Baron and Kenny, 1986). Hypothesis H1 was confirmed that proactive personality is negatively associated with migrant workers’ perception of technical unemployment risk. Proactive personality is positively associated with migrant workers’ AI learning (Model M2, *β* = 0.275, *p* < 0.001). With control applied on proactive personality, the relationship between AI learning and migrant workers’ perception of technical unemployment risk is insignificant (Model M3, *β* = −0.034, *p* > 0.05). Therefore, the mediating role of AI learning between proactive personality and migrant workers’ perception of technical unemployment risk is insignificant. Thus, Hypothesis H2 is not supported. This result indicates that although proactive personality can promote migrant workers to learn AI knowledge and skills, such learning behavior does not necessarily reduce their risk perception of technical unemployment, and the underlying reasons may lie in the effect of migrant workers’ learning, that is, whether learning can really help them cope with the impact of AI technology on their careers. Then, the mediating role of AI self-efficacy between proactive personality and the perception of technical unemployment risk was examined. Hypothesis H1 was confirmed that proactive personality is negatively associated with migrant workers’ perception of technical unemployment risk. Proactive personality is positively associated with the migrant workers’ AI self-efficacy (Model M4, *β* = 0.379, *p* < 0.001). With control applied on proactive personality, AI self-efficacy is negatively associated with migrant workers’ perception of technical unemployment risk (Model M5, *β* = −0.253, *p* < 0.001). That is to say, AI self-efficacy plays a significant mediating role between proactive personality and the perception of technical unemployment risk. Thus, Hypothesis H3 is supported. This result also confirms the view of the Social Cognitive Theory that migrant workers with proactive personality actively take actions to change their environment, so that they can have a stronger self-efficacy when dealing with the threats of AI to their career development. A stronger self-efficacy means that migrant workers continue taking actions to improve their environment, thereby further reducing the threats of AI to their career development and their perception of technical unemployment risk.

**Table 4:** Tests of the mediating effect of organizational green learning.

Paths	Effect Value	Standard Error	95% confidence Interval	
			Lower limit	Upper limit
X→M1→Y	0.0074	0.0275	−0.0458	0.0635
X→M2→Y	−0.1519	0.0415	−0.2377	−0.0768
X→M1→M2→Y	−0.0243	0.0096	−0.0464	−0.0090

Note: X is proactive personality; Y is perception of technical unemployment risk; M1 is AI learning; M2 is AI self-efficacy.

Then, SPSS PROCESS macro 3.4 was used to verify the mediating role of AI learning and AI self-efficacy again, the results of which are shown in Table 4. The analytical results show that the indirect effect of proactive personality on migrant workers’ perception of technical unemployment risk through AI learning is 0.0074 and 95% CI = [−0.0458, 0.0635], including 0. This result suggests that AI learning plays a insignificant mediating role between proactive personality and migrant workers’ perception of technical unemployment risk. Thus, Hypothesis H2 is not supported again. The indirect effect of proactive personality on migrant workers’ technical unemployment risk perception through AI self-efficacy is −0.1519, and 95%CI = [−0.2377, −0.0768], excluding 0. This result suggests that AI self-efficacy plays a significant mediating role between proactive personality and migrant workers’ perception of technical unemployment risk. Thus, Hypothesis H3 is supported again.

Finally, SPSS PROCESS macro 3.4 was used to verify the chain-mediating role of AI learning and AI self-efficacy, the results of which are shown in Table 4. The analytical results show that the indirect effect of proactive personality on migrant workers’ perception of technical unemployment risk through AI learning and AI self-efficacy is −0.0243, and 95%CI = [−0.0464, −0.0090], excluding 0. This result shows that AI learning and AI self-efficacy play a chain-mediating role between proactive personality and migrant workers’ perception of technical unemployment risk. Therefore, Hypothesis H4 is supported. Considering that Hypothesis H2 has not been supported, it can be shown that under the threat of AI technology, proactive personality can promote migrant workers to actively learn AI-related knowledge and skills, but it cannot directly reduce their perception of technical unemployment risk. Only when migrant workers significantly improve their self-efficacy in coping with the AI technology through AI learning, will they continue taking actions to change their unfavorable situations, thereby reducing their perception of technical unemployment risk.

## 5 Discussion

The present study examines how proactive personality is associated with migrant workers’ perception of technical unemployment risk and ascertains whether AI learning and AI self-efficacy play a mediating role or a chain mediating role between proactive personality and migrant workers’ perception of technical unemployment risk. Firstly, proactive personality is negatively associated with migrant workers’ perception of technical unemployment risk. Although no studies have directly explored the relationship between them, some studies have shown that proactive personality can improve individuals’ perceived employability (Guilbert et al., 2018). A high perceived employability means a low perception of technical unemployment risk. Therefore, the research in this paper is basically consistent with this conclusion. Secondly, proactive personality is positively correlated with AI learning, but there is no significant correlation between AI learning and technological unemployment risk perception. Therefore, AI learning does not play a mediating role between proactive personality and migrant workers’ perception of technical unemployment risk. Studies have shown that proactive personality can be used to actively predict individuals’ pursuit of skill improvement and participation in various trainings events (Guilbert et al., 2018; Brown et al., 2006). The research in this paper is consistent with these views. To counter the threats of AI to career development, individuals need to change their knowledg and skill structures in time (Wang et al., 2024). AI learning is an important way to acquire such knowledge and skills (Kuznia et al., 2010). However, AI learning does not reduce the risk perception of technological unemployment. The reason behind this may be that individuals may not accumulate relevant knowledge or skills to help them cope with the threats brought by AI after AI learning, which depends on their learning efficiency. Migrant workers’ learning of AI-related knowledge is a self-directed learning behavior. Regarding self-directed learning behavior, individuals need self-control in the adjustment of learning plans and processes (Dong et al., 2022). Therefore, AI learning requires individuals to have the ability of self-control (Liu, 2024), which will affect their learning effect (Zheng and Yu, 2022). Therefore, the mere learning of AI by migrant workers does not ensure the mastery of AI knowledge and skills, and it cannot ensure the reduction of perception of technical unemployment risk. Thirdly, the research in this paper shows that proactive personality has a significant positive correlation with AI efficacy, while AI efficacy has a significant negative correlation with the perception of technical unemployment risk. Therefore, AI efficacy plays a mediating role between proactive personality and migrant workers’ perception of technical unemployment risk. This finding is consistent with studies that, for example, on positive relationships between proactive personality and self-efficacy (Seibert et al., 1999; Shang and Gan, 2009; Li et al., 2017). At the same time, it also confirms the view of Social Cognition Theory (Bandura and Locke, 2003), AI self-efficacy improves the initiative of migrant workers and helps them to actively take measures to change their status quo, thus reducing the threat of AI to their career development, and having a lower perception of technical unemployment risk. Fourthly, the study has also found that AI learning and AI self-efficacy play a chain mediating role between proactive personality and migrant workers’ perception of technical unemployment risk. This conclusion suggests that a proactive personality prompts migrant workers to learn AI-related knowledge and skills, and then further improve the level of AI self-efficacy, which alleviates migrant workers’ perception of technical unemployment risk. Only when migrant workers have the self-control ability and master relevant knowledge and skills through learning, or recognize and imitate models in the process of learning, can they have a sense of self-efficacy in dealing with AI, thus reducing the perception of technical unemployment risk.

### 5.1 Theoretical implications

Based on the social cognitive theory, the relationship between proactive personality and migrant workers’ perception of technical unemployment risk was discussed in this paper. Also, the mediating role of AI learning and AI self-efficacy was explored. The main theoretical significance of this study is reflected in the following three points.

Firstly, regarding the impact of AI technology on employees, the existing studies have analyzed the unemployment risks facing female librarians in libraries and programmers (Zhu and Luo, 2021; Tang and Huang, 2024). There are few studies paying attention to the perception of technical unemployment risk of migrant workers under the impact of AI. Migrant workers are a vulnerable group in the society due to their low level of human capital, and their ability to resist unemployment risk is weak (Yang and Shao, 2021). Therefore, focusing the relevant research on the impact of AI on the employment of migrant workers can deepen the understanding of the special impact that AI has on various social groups, which is conducive to formulating targeted policies to intervene in the negative effects of AI on the employment of different groups.

Secondly, it enriches the research on the influencing factors of migrant workers’ perception of technical unemployment risk under the impact of AI. At present, there have been discussions conducted on the influencing factors of employees’ unemployment risk perception under the impact of AI. The focus of these discussions is placed on the impact of age, income, education level, professional field, employment type and industry (Wang et al., 2021). In addition, some scholars pay attention to the influencing factors of job insecurity under the impact of AI, mainly focusing on the factor of AI awareness (Lingmont and Alexiou, 2020). Based on the social cognitive theory, this paper discuss the impact of proactive personality, revealing their significant negative impact on the perception of technical unemployment risk of migrant workers. This enriches people’s cognition of the influencing factors of the perception of technical unemployment risk among migrant workers.

Thirdly, it deepens people’s understanding as to the mechanism of how the perception of technical unemployment risk is formed among migrant workers under the impact of AI. Although previous studies have explored the influencing factors of workers’ unemployment risk perception under the impact of AI technology (Wang et al., 2021), few studies have explored the formation mechanism of migrant workers’ technical unemployment risk perception. Based on the social cognitive theory, a discussion was conducted in this paper about the mediating role of AI learning and AI efficacy in the relationship between proactive personality and migrant workers’ perception of technical unemployment risk. This deepens people’s understanding of the formation mechanism of migrant workers’ perception of technical unemployment risk. At the same time, the conclusions of this research also provide a theoretical basis for organizing the formulation of policies to reduce migrant workers’ perception of technical unemployment risk.

### 5.2 Management implications

The empirical research results of this paper provide some implications for enterprises to alleviate migrant workers’ perception of technical unemployment risk, improve their job security and mental health, and thus implement the reform of AI successfully.

Firstly, the initiative of migrant workers to cope with the impact of the AI technology should be improved. The empirical results show that proactive personality is related to migrant workers’ perception of technical unemployment risk. Given the impact of AI on careers, if individuals can actively respond to this impact, they will have a lower perception of technological unemployment risk. First of all, from the perspective of migrant workers, they need to correctly understand and take proactive measures to deal with the impact caused by developing AI technology on their careers. Next，companies can implement some specific strategies to improve the initiative of migrant workers to cope with the impact of AI technology. For example, companies can create incentive policies that give material or spiritual rewards to employees who take actions to proactively respond to AI threats, thereby motivating them. In the end, from the perspective of an organization, when employees are recruited, selecting migrant workers with strong proactive personality traits by means of personality measurement tools is conducive to maintaining migrant workers’ perception of technical unemployment risk at a low level.

Secondly, it is necessary to guide migrant workers to actively learn AI-related knowledge and skills, as well as to improve their self-efficacy in coping with the threats of the AI technology. It has been found out that AI learning does not play a mediating role between proactive personality and migrant workers’ perception of technical unemployment risk. However, AI self-efficacy can mediate the relationship between them. Additionally, AI learning and AI efficacy can play a chain-mediating role in the relationship between proactive personality and the perception of technical unemployment risk. This conclusion shows that organizations need to improve the AI self-efficacy of migrant workers, which specially need to guide migrant workers to improve their self-efficacy in coping with AI through learning. According to Social Cognitive Theory (Wood and Bandura, 1989), there are four principal ways to strengthen AI self-efficacy, such as mastery experiences, through modeling, social persuasion and enhance their physical status. Specifically, firstly, organizations can also strengthen the construction of learning culture and guide migrant workers to actively learn AI-related knowledge and skills. For example, various study groups should be established within an organization to guide migrant workers on how to exchange and learn with each other and share learning experiences. Managers should take the lead in learning AI-related knowledge and skills, driving a learning atmosphere within the organization. When these policies and measures are implemented, it is necessary to regularly evaluate the learning effect of migrant workers and give timely feedback, so as to help migrant workers understand and improve their mastery of AI knowledge and skills. Secondly, migrant workers who have successfully changed their knowledge and skills structures should be set up as models, so that others’ self-efficacy can be improved by imitating and learning from these role models. Thirdly, enterprises need to strengthen the publicity of the AI technology among migrant workers to correctly understand changes caused by implementing the AI technology in the working environment and the opportunities presented for individuals in this process. Through publicity and encouragement, help migrant workers build confidence in dealing with the AI technology. Fourthly, enterprises set up various fitness facilities and psychological counseling institutions to help employees enhance their physical status and to reduce their stress levels, which is beneficial for migrant workers to truly improve their sense of efficacy in coping with AI through learning.

### 5.3 Limitations and future directions of research

The limitations of this study mainly include the following aspects.

Firstly, the data source of this paper is comprised mainly of the self-reports of migrant workers and the data used in this paper are cross-sectional data, which cause the common method variance to a certain extent and makes it difficult to confirm the causal relationship between the variables. In addition, this article may also have endogeneity problems, such as omitted variables and simultaneity effects (i.e., reverse causation). In the future, some measures can be taken to ameliorate these problems and thus improve the effectiveness of this study. Firstly, some variables, such as AI learning, can be obtained through third-party evaluation to reduce the interferences of common method variance. Secondly, the method of experiment methods can be considered to further verify the causal relationship among these variables in the future. Meanwhile, it also can effectively eliminate endogeneity and CMV (Cooper et al., 2020). Thirdly, according to the econometric method, we will select appropriate instrumental variables and obtain corresponding data in future studies to reduce the influence of endogeneity.
Secondly, the AI learning variable used in this paper is only measured using a comprehensive question, which may not adequately capture the complexity of the concept. In the future, a more comprehensive scale can be considered for measurement, such as, employee engagement in learning activities (Bezuijen et al., 2010) and career-related continuous learning (Kuznia et al., 2010).
Thirdly, this paper only examines the mediating and chain mediating effect of AI learning and AI self-efficacy, with no attention paid to examining the boundary conditions in which proactive personality influences migrant workers’ perception of technical unemployment risk. The Social Cognitive Theory holds that human activities are determined by behavior, cognitive and other personal factors, and environmental events (Wood and Bandura, 1989). In other words, both personality and environment shape individual behavior. Some studies have found that supervisor support for training reflects that enterprises attach importance to employees’ personal development, which will have a positive impact on employees’ developmental need awareness, motivations to learn and transfer, as well as job performance (Park et al., 2018). In the context of AI development, supervisor support for training may strengthen the influence of proactive personality on employees’ AI learning behavior, AI self-efficacy and perception of technical unemployment risk. Therefore, in the future, the moderating role played by supervisor support for training in the relationship between proactive personality and migrant workers’ perception of technical unemployment risk is worth further exploring. At the same time, there are significant differences in the education level, learning willingness and development demands of migrant workers of different generations (Du and Zhang, 2008), Besides, their cognition and coping ability to the impact of AI technology are also bound to differ. In the future, it is suggested to continue examining the moderating role of generations in the relationship between proactive personality and migrant workers’ perception of technical unemployment risk.
Fourthly, some studies have found that the substitution risks of AI for workers in different occupations or sectors are not consistent (Wang et al., 2022). This study does not distinguish the occupations or sectors of samples. Therefore, future studies may consider comparative studies on samples from different occupations or sectors.

## 6 Conclusion

The present study contributes the perception of technical unemployment risk literature by highlighting the impact of proactive personality and the chain mediating role of AI learning and AI self-efficacy in a sample of Chinese migrant workers. The findings illustrated the influence of proactive personality on migrant workers’ perception of technical unemployment risk and confirmed AI learning and AI self-efficacy mediated this relationship independently, and played a chain-mediating role in this relationship. A key strategy to reduce migrant workers’ perceptions of technological unemployment risk is to improve migrant workers’ initiative in managing the impacts of AI technology, particularly by encouraging migrant workers to engage in AI learning to improve their self-efficacy against AI threats.

## Data Availability

The raw data supporting the conclusions of this article will be made available by the authors, without undue reservation.
